# Long-Term Use of Ruxolitinib in an AML Patient with Posttransplant Steroid Refractory GVHD

**DOI:** 10.1155/2020/4936846

**Published:** 2020-04-06

**Authors:** Philip T. Sobash, Achuta K. Guddati, Vamsi Kota

**Affiliations:** ^1^Internal Medicine, White River Health System, Batesville, AR, USA; ^2^Medical Oncology, Augusta University, Augusta, GA, USA

## Abstract

Ruxolitinib has become a new therapeutic option for steroid refractory graft-versus-host disease (srGVHD), with a substantial remission rate. Its anti-inflammatory properties by blocking interleukin pathways have made it a novel therapeutic approach to inflammatory disease processes, such as GVHD. The long-term use of ruxolitinib has not been explicitly studied outside the context in the treatment of multiple myeloma. With current clinical trials underway for the use of ruxolitinib in srGVHD, there are still no current guidelines or protocols for long-term clinical use. Of the available literature showing ruxolitinib utilization for srGVHD, most cases lead to resolution and eventual discontinuation. We present a case of a 32-year-old male on ruxolitinib with GVHD status postmatched unrelated donor stem cell transplant (MUD SCT) for acute myeloid leukemia (AML) with FLT3 mutation currently on ruxolitinib for 5 years who is not able to tolerate reduction in dosage due to flare-ups. We discuss the clinical implications and nuance of therapy with ruxolitinib with unknown long-term effects and weigh the risks and benefits.

## 1. Introduction

Steroid refractory GVHD (srGVHD) has a poor prognosis, with survival rates between 5 and 30% [1]. Ruxolitinib has been shown to reduce side effects from srGVHD, in both acute and chronic settings, with some studies showing 81.5% overall response in an acute state and 46.3% in a chronic state [2]. Responses have been shown to occur mostly early in treatment with a 68% discontinuation rate and 21% reduction in steroid use [3]. Multiple centers have noted the promising effects [2, 3] along with clinical trials underway [4, 5].

Many of the best available treatments for srGVHD with current immunomodulatory drugs have side effects including lymphoproliferative disorders, relapse of disease, cancer, and opportunistic infections. While there have been reports of cytomegalovirus infection and pancytopenias on ruxolitinib [2, 6], others did not show these effects [3]. As dosages and treatments were similar, it is hard to speculate why there was a discrepancy between data sets. It is potentially due to the differing use of steroids and other immunosuppressive agents in conjunction with ruxolitinib. Further studies with sole administration of ruxolitinib are needed to determine if it is a causative agent for side effects or if there are other confounding variables.

To the authors' knowledge, no studies have assessed the use of ruxolitinib in terms of long-term sequela. Many effects have been noted in the treatment of sr-acute GVHD, but less for sr-chronic GVHD due to lack of necessity for further therapy with reduction of symptoms. Prolonged use of therapy due to inability to taper has not been specifically studied. This is due in part to the high percentage of patients who respond and ultimately are discontinued from therapy. Herein, we report our experience with a patient who responded to ruxolitinib and has been on the drug for 6 years with flare-ups despite prolonged taper schedule.

## 2. Presentation

A 26-year-old patient presented to an outside facility in late 2013 with a WBC count of 260,000, subsequently diagnosed with AML with FLT-3 mutation, and started on emergent treatment. He was initially treated with 2 rounds of induction therapy with 7 + 3 regimen, but his disease state remained persistent. Given his young age and previous history of mucositis, pneumonia with sepsis, and a decreased ejection fraction due to previous therapy, he was given a course of high-dose cytarabine 1500 mg/m^2^ with sorafenib. Ultimately, he did receive a MUD SCT with fludarabine/busulfan (4)/antithymocyte globulin regimen in November 2013. Immunosuppressive regimen consisted of tacrolimus and methotrexate. The patient began displaying mild aGVHD after transplant and received topical steroids. Bone marrow biopsy 30 days from transplant showed minimal residual disease (MRD) and FLT3 positivity on PCR. The patient was then started on sorafenib 200 mg BID and azacytidine and continued on GM-CSF to help with GVHD with plans to rebiopsy in one month. At this time, a second transplant was discussed but ultimately decided to not be an option due to organ problems prior to the transplant. A repeat bone marrow biopsy in February/2014 showed no activity of disease indicating hematological and morphological remission. Patient received azacytidine for 4 days at 75 mg/m^2^ and was given a donor lymphocyte infusion (DLI) in March/2014. Although he continued to be in remission since that DLI, he had a flare-up of GVHD post-DLI. He presented with severe chronic GVHD involving the skin, eyes, and lungs in May 2014. Skin manifestations included rapidly progressing sclerotic appearance typically seen in the chronic GVHD setting and involving more than 50% of his body surface. Lung GVHD was diagnosed with follow-up pulmonary function tests where his DLCO dropped to 20% of predicted. His immunosuppression at the time of his presentation consisted of tacrolimus alone with levels being between 5 and 7 ng/ml as it was being tapered slowly and levels kept lower due to his high risk of relapse.

He initiated therapy with prednisone at 2 mg/kg given his severe GVHD (NIH severity 4) in addition to increase in tacrolimus dosing to achieve levels in the 7-10 ng/ml range. He did have a response to the high-dose steroids but rapidly developed complications of steroids therapy most notably, steroid myopathy (CTCAE v4.03, grade 3) involving both the upper and lower limbs in the typical proximal muscle distribution seen with steroids. On high-dose steroids, his GVHD symptoms did improve with skin GVHD having a partial response and improvement in his DLCO to 40%. Attempts to taper to below 20 mg resulted in a flare-up of skin GVHD as well as worsening of lung function. He also started having progressive skin symptoms despite being on steroids, and mycophenolate mofetil (MMF) was added to his immunosuppressive regimen. He did not have any benefit in terms of either clinical improvement of GVHD or reduction in steroid dose, and so MMF was discontinued and he had an increase in steroid dose. Ruxolitinib 5 mg PO BID was started in 2014, when steroid therapy proved to be ineffective, and he had an excellent rapid response to symptoms. His skin GVHD was controlled with improvement appearing within the first month of initiation of therapy. His lung function also stabilized with DLCO in the 35-45 range. He was weaned off of other immunosuppressants quickly at the patient discretion but did not have a flare-up despite a rapid taper of steroids. His myopathy resolved as expected within the first month after discontinuation of steroids. At present time, his cGVHD is under control with ruxolitinib alone. Attempts to wean off of his current dosage of ruxolitinib have proved unsuccessful as cGVHD flares up and symptoms worsen. His weaning attempts initially were per standard recommendations with the dose tapered to 5 mg once a day and plans for discontinuation. He was never off ruxolitinib for prolonged periods as his symptoms flared up within a week and had to restart therapy. His GVHD did respond to restarting the ruxolitinib at 5 mg twice a day. Patient seemingly tolerates the treatment with ruxolitinib, and he has had no identifiable issues or side effects from long-term use. Of note, he did have one admission to the hospital for community-acquired pneumonia in 2018 which resolved with appropriate antibiotic therapy. The current prospective treatment plan involves a taper attempt every 3-4 months with 2.5 mg every two months to wean off therapy as tolerated. The clinical question then becomes if a patient is seemingly gaining benefit from ruxolitinib therapy, does the risk of the unknown long-term therapy use outweigh the benefits of current stability of suppressing cGVHD.

## 3. Discussion

Given the relatively recent utilization of ruxolitinib for srGVHD and the varying responses, the proper management of patients on long-term therapy in the absence of clinical guidelines is unclear. To date, no long-term toxicities have been noted, although some infections and viral reactivations have been seen as noted earlier. In our experience with this patient, we have only identified one potential negative event in terms of infection in an event of pneumonia. Even then, this could be due to multiple factors and not directly attributed to ruxolitinib, although this cannot be determined. The authors of this paper speculate though that like any long-term immunosuppressive, clinical judgement should be used to determine a risk/benefit analysis. In our specific case, tapering causes severe GVHD flare-ups and hence the reason for continued use. With no seemingly therapeutic consequences of long-term use, we argue that there may be no reason not to continue our patient on therapy and monitor closely as with other immunosuppressive therapies.

Another consideration is the mechanism of action of ruxolitinib versus other immunomodulating drugs. While both are acting on regulation and proliferation of T-cells, ruxolitnib acts on the Jak1/2 kinase pathways. The exact impact on T-cell regulation and proliferation is still poorly understood [7]. For example, in some studies, ruxolitinib decreased regulatory T-cells while in others, it increased [8]. There may be more of an involvement in reduction of inflammatory cytokines rather than absolute T-cell numbers compared to calcineurin inhibitors.

There have been multiple studies highlighting the long-term use of ruxolitinib in individuals with myelofibrosis with side effects including pancytopenias, viral reactivation, and infections. Liver toxicity with longer-term use in patients with myelofibrotic disorders has been reported [9]. While many studies were a different disease state, much of the length of therapy overlaps with our patient to some extent. It is reasonable then to state that the length of therapy in regard to our patient is in line with that in other uses for ruxolitinib in the context of long-term side effects. A final thought is that increased doses may harbor an unforeseen effect on the sr-cGVHD process. Most studies that have gone by the dosing parameters outlined by Zeiser *et al.* [2]. Our experience thus far has not shown any increased overall morbidity or mortality due to long-term ruxolitinib use. More studies and cases are needed to assess long-term outcomes of ruxolitinib use and if there are any side effects in the long-term, other than reported, to be weighed into management decisions.
